# Assessing Acceptability of a Diagnostic and Malaria Treatment Package Delivered by Community Health Workers in Malaria-Endemic Settings of Burkina Faso, Nigeria, and Uganda

**DOI:** 10.1093/cid/ciw630

**Published:** 2016-12-06

**Authors:** Ayodele S. Jegede, Frederick O. Oshiname, Armande K. Sanou, Jesca Nsungwa-Sabiiti, IkeOluwapo O. Ajayi, Mohamadou Siribié, Chinenye Afonne, Luc Sermé, Catherine O. Falade

**Affiliations:** 1 Department of Sociology, Faculty of the Social Sciences; 2 Department of Health Promotion and Education, Faculty of Public Health; 3 Department of Epidemiology and Medical Statistics; 4 Epidemiology and Biostatistics Research Unit, Institute of Advanced Medical Research and Training; 5 Department of Pharmacology and Therapeutics, College of Medicine, University of Ibadan, Nigeria; 6 Groupe de Recherche Action en Santé, Ouagadougou, Burkina Faso; 7 Child Health Division, Ministry of Health, Kampala, Uganda

**Keywords:** community health worker, malaria, acceptability of treatment, child health, ACT

## Abstract

***Background.*** The efficacy of artemisinin-based combination therapy (ACT) and rectal artesunate for severe malaria in children is proven. However, acceptability of a package of interventions that included use of malaria rapid diagnostic tests (RDTs), ACTs, and rectal artesunate when provided by community health workers (CHWs) is uncertain. This study assessed acceptability of use of CHWs for case management of malaria using RDTs, ACTs, and rectal artesunate.

***Methods.*** The study was carried out in Burkina Faso, Nigeria, and Uganda in 2015 toward the end of an intervention using CHWs to provide diagnosis and treatment. Focus group discussions (FGDs) and key informant interviews (KIIs) were conducted with parents of sick children, community leaders, and health workers to understand whether they accepted the package for case management of malaria using CHWs. Transcripts from FGDs and KII recordings were analyzed using content analysis. The findings were described, interpreted, and reported in the form of narratives.

***Results.*** Treatment of malaria using the CHWs was acceptable to caregivers and communities. The CHWs were perceived to be accessible, diligent, and effective. There were no physical, social, or cultural barriers to accessing the CHWs’ services. Respondents were extremely positive about the intervention and were concerned that CHWs had limited financial and nonfinancial incentives that would reduce their motivation and willingness to continue.

***Conclusions.*** Treatment of malaria using CHWs was fully accepted. CHWs should be compensated, trained, and well supervised.

***Clinical Trials Registration.*** ISRCTN13858170.

Effective case management of malaria entails clinical assessment and laboratory confirmation via light microscopy or rapid diagnostic tests (RDTs) prior to treatment with an effective antimalarial [1–4]. There is evidence that deploying artemisinin-based combination therapies (ACTs) and rectal artesunate at the community level using trained lay community members is feasible, effective, and saves lives [5, 6]. The use of RDTs in the community setting is increasing but far less studied [3, 7–11]. We could find some studies on the acceptability of deploying RDTs to confirm diagnosis of malaria in the community setting, either with RDTs and ACTs, or RDTs and rectal artesunate, but none with all 3 components, which is currently advocated [10, 12–14].

We therefore undertook a study to understand acceptability for the use of community health workers (CHWs) to provide an integrated intervention with RDTs, ACTs, and rectal artesunate in Burkina Faso, Nigeria, and Uganda. The countries were chosen because they are among the top 10 countries that contribute 80% of global malaria cases, and in the top 15 countries that contribute 78% of deaths from malaria [15]. Lay community members providing health services are called community medicine distributors in Nigeria, village health teams in Uganda, and Agents de Santé Communautaire in Burkina Faso, hereinafter referred to as CHWs. The study aimed to investigate whether such an integrated approach by CHWs is acceptable and whether there were any misgivings about any component of the intervention. We expected to find some concerns about the use of CHWs to take blood for RDTs [16], concerns about not providing treatment to children with an RDT-negative test, but acceptability of treatment with rectal artesunate for children with danger signs, and positive reviews of earlier case management of fever in young children in the participating countries.

## METHODS

### Study Site

In Nigeria, 33 randomly selected communities from 2 rural health districts of the Ona-Ara local government area (LGA) constituted the study sites. In Uganda, the study sites were Kayunga and Sheema, in the subcounties of Busaana and Kyangyenyi. In Burkina Faso, 45 villages were chosen in the health area of Sidéradougou, Mangodara District [17].

### Study Design

This qualitative study was 1 component of a quasi-experimental study carried out to evaluate a package for diagnosis and treatment of malaria of varying degrees of severity in 3 countries in sub-Saharan Africa [17]. After 1 year of intervention, during which ACTs were used for treatment of young children after a positive RDT diagnosis, the communities' perception of CHWs' activities was explored. Focus group discussions (FGDs) and key informant interviews (KIIs) were conducted.

### Conduct of Qualitative Study

In Nigeria, 4 FGDs and 10 KIIs were conducted with key stakeholders: caregivers, community leaders, opinion leaders, religious leaders, health workers, and the husbands of CHWs. The interviews were conducted by trained research assistants in 2 teams of 3 (moderator, notekeeper, and recorder) supervised by a social scientist on the research team. The research assistants had 3 days' training prior to study conduct. Training included familiarization with data collection instruments and practical sessions on interviewing skills—techniques of probing, recording responses, and note-taking, as well as transcription of recordings. Field guides were developed and pretested prior to use. The guides were translated into the local language, Yoruba. Participants at both the FGDs and KIIs were shown the tape recorder; verbal informed consent to record the sessions and interviews was obtained from each. The FGD sessions and KII lasted between 30 and 60 minutes. Interviews and FGDs were conducted to the point at which no new information was elicited. Transcription was completed daily by those conducting interviews.

In Uganda, orientation of the research team by the study investigator was carried out for 2 days during which tools were reviewed, pretested, and adopted. KIIs were conducted with CHWs, health workers, and local counselors attached to health centers in the study sites of Sheema and Kayunga districts. A total of 5 FGDs with 35 CHWs was carried out with 3 FGDs for male CHWs and 2 for female CHWs. Seven KIIs were carried out with clinical officers from Health Centre III and enrolled nurses from Health Centre II to obtain information on where communities access and receive treatment for malaria. Data were collected on malaria treatment-seeking and provision of services from KII respondents.

In Burkina Faso, 3 communities were randomly selected, 2 of which had a combined intervention of RDTs plus ACTs and rectal artesunate, and 1 with only RDTs and ACTs [17]. In each selected community, 2 FGDs were carried out (1 group of male caregivers and 1 group of female caregivers: 8 participants each), with participants identified by the CHWs. Caregivers selected were village residents, whose child had been treated for an illness episode within the preceding 6 months. The FGDs focused on participants' impressions of the interventions, perceived role of CHWs in providing access to care, perceived satisfaction quality of care, supervision, and drug supply.

### Analysis

The audio-recorded interviews and discussions were transcribed. Codes were developed and finalized by a single coder in each country. An integrated approach was used for the coding, making it both deductive and inductive. Deductive coding was conducted at the initial phase. The study objectives served as the basis of coding and titles of codes (nodes) in “tree nodes.” Following an inductive tradition, we were able to achieve “coding on.” Hence, content of initial nodes mainly determined the codes and code structure that were further developed. All interviewees were assigned anonymous respondent numbers.

### Ethical Approval

Ethical approval for the study was obtained from the Ethics Review Committee of the WHO; the Ethics Committee of the University of Ibadan/University College Hospital, National Health Research Committee, and Oyo State Ministry of Health in Nigeria; the National Ethics Committee for the Research on Health in Burkina Faso; and the National Council for Science and Technology in Uganda. Informed consent was obtained from the community and participants in the study.

## RESULTS

There were 3 key findings in this study: (1) CHWs were accepted and trusted in their communities because of the neutral way in which they managed patients based on the RDT results and because of the perceived effectiveness of the medicines used; (2) there was acceptance of their role as community health providers if they had adequate supervision and refresher training so that their skills were retained; (3) it was feared that the CHW program could not be sustained without better financial motivation, especially during periods when there was high demand for their time.

### Role of CHWs as Healthcare Providers

The overall response of the community was very positive. CHWs were reported to have played an important role in healthcare delivery and were perceived to be accessible physically and socially:

*The first  …  benefit of the program is that it relieves us of transport burden that used to be our major problem when it comes to taking a sick child to government hospitals  …You know in 2 hours you have not gotten there. We now take our children to them [CHWs] without collecting any payment and they take good care of the children. Secondly, any child taken to them is treated accordingly, there is no preference for selecting or rejecting a particular child, there is no discrimination or maltreatment or being rude to any parent. They answer us on time and take care of the children well. Third they treat our children, carry out blood tests on them, administer drugs to them, even visit our homes to check on them and inquire about their health status. Tell me which nurse or doctor would do that for you if you take your child to hospital. So there are lots of benefits from them.* — Male caregivers, FGD, Badeku, Nigeria

### Changes in Care-Seeking Behavior Because of the Program

Within the short period of the program, treatment-seeking behavior was reported to change. Care-seeking for fever or danger signs in children before the program was largely self-medication, purchases from street vendors or drug shops, traditional healers, and health centers. In Nigeria, respondents affirmed that caregivers of children are used to giving their children all sorts of drugs in a combined form. The change was noted in all countries:

*Before this study some went to the health center. Others made of self-medication including paracetamol. Some bought the drugs in hawkers*. — Community leader, KII, Gouandougou, Burkina Faso

*Our fathers taught about the use of herbal drugs for children when they are sick. These were the same drugs that they use to raise us. They taught us how to use and make the herbal drugs. We used to give these herbal drugs to our children when they are sick. It is when this program started that we now give modern medicine to our children who have malaria.* — Male caregivers, FGD, Akanran, Nigeria

*As for these cases especially convulsions, they say the child has “yabwe” (fits)  …  They first use local herbs to cleanse the child [from] bad spirits and rush to the shrines to traditional healers to help save the child's life.* — Male CHWs, FGD, Nakatovu, Uganda

Respondents commented that people became more enlightened and educated about malaria. In Uganda particularly, CHWs confirmed that caregivers came to them, and attributed this to the ease of geographical access, availability of medicine stocks, and the awareness of communities. In the female FGDs, the reasons for consulting other types and forms of healthcare ranged from cultural and religious beliefs, comorbidity, or symptoms suggestive of other diseases, to lack of awareness about the presence of CHWs. All male FGD participants affirmed that some community members were skeptical about the ability of CHWs to provide malaria treatment, as CHWs were not known for such practices in the past. Discussants commented on the free healthcare and easy accessibility of the CHWs:

*I don't take my children to any other place rather than the CHWs, since these people are near us and living among us, they treat our children well. Unlike the Government health center where we would still spend 3 hours taking transport  …  and when we get there they will still behave anyhow [discourteously] despite the fact that we are still going to pay for the treatment*. — Male caregivers, FGD, Akanran, Nigeria

*Many caregivers  …  fear that they are not well trained like registered nurses. We are trying to educate them that they are trained to treat malaria in children*. — Male caregivers, FGD, Badeku, Nigeria

### Strengths of CHWs in the Study Communities

Immediate physical access, social accessibility, and effective medications were major factors for the utilization of CHWs. A high proportion of discussants reported that the CHWs are always available when needed, were friendly, and were ready to treat children without cost:

*What makes them [the CHWs]  …  different is that they are closer  …  They attend to us anytime  …  Once they test the child and she is reactive to the malaria test, they will attend to her immediately. They are very friendly. They are accommodating. Once you get there, they will collect the child and conduct blood tests with one glass-like instrument. Once the child is positive, they will give us drugs. The drugs are also very effective  …  [the drugs] work wonders  …  like magic*. — Female caregivers, FGD, Badeku, Nigeria

*When I took my child to the CHW, she was very welcoming. She collected my baby from me and showered her because the baby had high temperature and observed for about 5 minutes. After this, the CHW did blood tests and gave us drug. In the evening, she checked on the baby to ask after her health. Early next morning, she came to check on the baby again. Summarily, they are very accommodating, warm, friendly, nice and welcoming.* — Female caregivers, FGD, Akanran, Nigeria

### Reduced Cost of Treatment as an Additional Major Benefit

*The CHWs are viewed as health workers because they are close to us and have very effective treatment for our children. It is difficult to get to health centres. The transportation costs alone is 1000 FCA for return trip aside from the cost of prescriptions, consultation, and money to buy food. But with CHWs it makes life much easier.* — Women, KII, Djalakoro, Burkina Faso

### Weaknesses of the CHW Program

Generally, respondents revealed no negative aspect of the CHW program; indeed, many respondents solicited greater CHW empowerment:

*I do not see anything that needs to be corrected. There is no shortcoming or weakness. They are doing their work very well. They are very close to us. Anytime we get there, they answer us very well. I do not see anything to correct in what they are doing. All that I can say is that you should empower them more.* — Female caregivers, FGD, Akanran Axis, Nigeria

*I do not see any negative aspect in the presence of CHWs within our community. It's the end of the project which is a problem for them as parents/guardians think that it is the CHWs who want to stop the project while the benefits of the drug are enormous.* — Community leader, KII, Sidéradougou, Burkina Faso

The only negative point made was the desire to extend the program to older children and to adults. Some said that if adults are not well, they cannot care for the children.

### Caregivers' Perception of RDTs

Respondents were also impressed with the methodology involved in testing the sick children. In Nigeria, community members had no misgivings about RDTs, although they mentioned that reticence in accepting the use of RDT relates to cultural and religious practices and fears of contracting other infections, and they mentioned specific ethnic groups as not accepting RDTs or modern medicine. Otherwise, it was noted that there was always consent for an RDT, although some stated that caregivers may not trust the CHWs’ competence to conduct or interpret RDTs:

*The benefit we see there is that they will first do test before they give any treatment. They will not just prescribe drugs based on the symptoms the child has. They have good attitude.  …  They are available and accessible to us.* — Female caregivers, FGD, Badeku, Nigeria

*They go complaining to other community people that we tested a child and it had no malaria yet when they went to the microscope [malaria] was found.* — Male CHW, FGD, Kyangyenyi, Uganda

### Factors Regarding Acceptance of Rectal Artesunate

Rectal artesunate was perceived as a beneficial means of protecting or saving children, with a positive view on the effectiveness of the drug as prereferral treatment for children with danger signs, aided by CHWs’ links with local health staff, and their own easy relationships with CHWs:

*When the child was taken to the CHW, she was almost lifeless and it was the [drug] that sustained the child till they took her to Tofunmi Hospital. Even when we got to the hospital, they could not find the right blood for her and we had to wait for a while before they could get the right blood type  …  They said if it was not for the [drug], the child could have died.* — Male caregivers, FGD, Akanran, Nigeria

*Some time ago, I observed that immediately a child was brought to a CHW and she found that the child had severe malaria, the CHW inserted the rectal artesunate into her anus and referred the child to a hospital in Ibadan.  … In the case of the child that I was talking about, the child was admitted in the hospital, treated and discharged on the third day. We thank the project managers so much, had it been the olden days, the child would have died. The drug inserted into the anus is like a magic, once used, it works well. We want the program to continue.* — Male caregivers, FGD, Akaran, Nigeria

### Community Satisfaction and Benefits

Participants revealed that the community members had benefited from the services provided by the CHWs. Most respondents appreciated improved, affordable, and easy access to healthcare for children in the community:

*In my village people used to go to Masheruka private clinics  …  People now save time for treatment because they get the treatment from the [CHW]… in the  …  village and run back to their gardens.* — Male CHWs, FGD, Kyangyenyi, Uganda

*They treat our children even if we go to them in the middle of the night without any complaint, without collecting any money from us  …  We benefit a lot*. — Male caregiver, FGD, Akanran, Nigeria

Most respondents in Nigeria and Burkina Faso agreed that the CHWs serve as a link between the communities and health center and that the CHWs appeared to have good relationships with the health centers:

*CHWs are seen as health workers. They represent a link between community and health center. They also improve access to health services because their presence is of great use.* — Head, Gouandougou community, KII, Burkina Faso

### Challenges Faced by CHWs

A range of challenges facing the CHWs was mentioned. These were inadequate logistics, financial burdens, workload, difficulties of referring patients, and limited involvement of males. It was observed that when CHWs do not have adequate supplies, trust in the program will be eroded and result in lack of patronage by the caregivers, who go where there are available supplies:

*If the drug is with one CHW and not with another, it could cause friction  …  as people will patronise the CHW with the drugs or kits. You know that not all of them  …  have drugs always*. — Female, FGD, Badeku, Nigeria

Respondents pointed out (mainly in Nigeria) that a major challenge facing the CHWs is finances. Because the CHWs are not paid any allowances, it would be difficult for them to travel to see the child:

*The number one challenge faced by the CHWs is finance as their movement from one village to another might be difficult because of the cost of transportation. When caregivers who stay in far villages contact the CHWs for the treatment of their children who have malaria, the CHWs may complain of money for bus or okada “bike” to transport them to the sick children's place and  …  lament not being paid.* — Nurse, head of PHC, Badeku, Nigeria

In Uganda, concerns were about the limited medicines given to CHWs and the workload:

*Most of the [CHWs] complain about leaving work they have to do behind. Some say their husbands are discouraging them to work as [CHWs] because they don't earn anything in return  …  [CHWs]  …  also have no torches, solar lights at night, gumboots, umbrellas, no bicycles.* — Health Worker (HW), KII, Rushozi, Uganda

### Suggestions for Improvement

Respondents were asked to suggest how to improve the program in the communities. Most respondents suggested increasing the number of CHWs in each community, providing training on communication, creating awareness, providing supervision, and motivating CHWs to function well. It was argued that there were too few CHWs for the number of communities being served; hence, all respondents unanimously agreed that more people should be recruited and trained as CHWs:

*Increases in the number of CHWs  …  will help the workload  …  and caregivers will have more access to them.* — Community leader, KII, Sidéradougou, Burkina Faso

Respondents across the 3 countries, including CHWs, considered that they should be trained regularly to improve their skills, and supervised:

*… refresh the skills of CHWs on managing sick children, on interpretation of RDT results on diagnosis of fast breathing (counting breathing rates), most CHWs think it is pneumonia. Teach others to seek care earlier when they notice any symptoms in their children to avoid them ending up with severe conditions [because] managing referral and danger signs is a challenge when they [cannot] convince the caregiver to take the child to the facility.* — HW, KII, Busaana, Uganda

*Supervision is to ensure quality of service delivery. When people are not supervised they will do what they want.* — Community leader, KII, Sidéradougou, Burkina Faso

It was generally agreed that the CHWs should be paid either centrally, through individual transactions, or through nonmonetary incentives:

*Motivating the CHWs by giving them gumboots, rain coats, and at least a monthly wage of Shillings 100 000 [USD $28]* *and refresher training  …  so that they don't forget what they learnt.* — HW, KII, Busaana, Uganda

*The CHWs can be  …  celebrated  …  in the community. Communities can give them special recognition and exempt them from some communal work  …  as a mark of recognition.* — Male caregiver, FGD, Akanran, Nigeria

There was strong support for continuity of the project.

## DISCUSSION

Malaria is an important cause of morbidity and mortality in children, and prompt access to diagnosis and care decreases the risk that the disease evolves to severe malaria and death [18, 19]. This study evaluated community response to a program delivering a package of diagnosis and treatment via CHWs in 3 countries in sub-Saharan Africa [17]. Caregivers and community members provided overwhelming support to the package of services provided. Immediate access to CHWs at little or no cost and rapid resolution of illness contributed to community acceptance. Stakeholders were impressed with the geographical and social access to CHWs, the systematic diagnosis provided, and perceived effectiveness of the medicines being used. Our findings are consistent with those of other studies [10, 20]. It was reported that the CHWs were effective in treating and following up treated cases and were able to successfully bridge the gap that normally exists between health facility workers and community members. There was no mention of any change in workload at the health facilities.

To a large extent, caregivers appeared to have confidence in the competence of the CHWs in performing RDTs and administering medication, but commented that there should continue to be refresher training and supervision to maintain quality. Retraining was important to refresh skills, provide opportunities to improve, and facilitate interactions between CHWs and formal healthcare workers who were sometimes critical of CHW performance and competence. There were many comments on the diligence with which the RDTs were used; as a result, and contrary to other studies where community members reject collection of blood due to lack of trust of use of blood samples, caregivers did not appear to be averse to the RDT for malaria [10]. Such was the acceptability of the program that respondents began to worry about methods for making the program sustainable, asked that the program be extended to other age groups, and suggested increasing the number of CHWs to reach more communities and to reduce the workload for each volunteer.

Inadequate incentives for volunteer CHWs was a leitmotif in discussions. A conflict of interest between personal work and the volunteer service was expected to hinder effective performance in resource-poor, rural settings, without financial incentives for the volunteers. Effective motivation was also commonly mentioned as a major challenge that would influence sustainability of the diagnostic and treatment program. Although there was no consensus on what financial strategies would be practical to sustain the program, there was agreement that incentives were essential, although strategies in one country may not be relevant elsewhere. While many suggested monetary incentives, others considered that nonmonetary incentives such as special recognition by their communities would be an important beginning to reduce CHW attrition rates [5, 21].

The package of interventions for malaria case management was welcomed, and the role of the CHWs in its delivery was appreciated. Participants valued getting malaria treatment closer to their homes and modified their treatment-seeking behavior in consequence.

## References

[1] Uzochukwu BSC, Onwujekwe E, Ezuma NN, Ezeoke OP, Ajuba MO, Sibeudu FT Improving rational treatment of malaria: perceptions and influence of RDTs on prescribing behaviour of health workers in southeast Nigeria. PLoS One 2011; 6:e14627. 21297938 10.1371/journal.pone.0014627 PMC3031496

[2] Ansah EK, Narh-Bana S, Epokor M et al Rapid testing for malaria in settings where microscopy is available and peripheral clinics where only presumptive treatment is available: a randomised controlled trial in Ghana. BMJ 2010; 340:c930. 20207689 10.1136/bmj.c930 PMC2833239

[3] Skarbinski J, Ouma PO, Causer LM et al Effect of malaria rapid diagnostic tests on the management of uncomplicated malaria with artemether-lumefantrine in Kenya: a cluster randomized trial. Am J Trop Med Hyg 2009; 80:919–26. 19478249

[4] World Health Organization. Guidelines for the treatment of malaria. 3rd ed Geneva, Switzerland: WHO, 2015.

[5] Ajayi IO, Jegede AS, Falade CO, Sommerfeld J Assessing resources for implementing a community directed intervention (CDI) strategy in delivering multiple health interventions in urban poor communities in southwestern Nigeria: a qualitative study. Infect Dis Poverty 2013; 2:25. 24156481 10.1186/2049-9957-2-25 PMC4177198

[6] Gomes MF, Faiz MA, Gyapong JO et al Pre-referral rectal artesunate to prevent death and disability in severe malaria: a placebo-controlled trial. Lancet 2009; 373:557–66. 19059639 10.1016/S0140-6736(08)61734-1 PMC2646124

[7] Altaras R, Nuwa A, Agaba B, Streat E, Tibenderana JK, Strachan CE Why do health workers give anti–malarials to patients with negative rapid test results? A qualitative study at rural health facilities in western Uganda. Malar J 2016; 15:23. 26754484 10.1186/s12936-015-1020-9 PMC4709931

[8] D'Acremont V, Kahama-Maro J, Swai N, Mtasiwa D, Genton B, Lengeler C Reduction of anti-malarial consumption after rapid diagnostic tests implementation in Dar es Salaam: a before-after and cluster randomized controlled study. Malar J 2011; 10:107. 21529365 10.1186/1475-2875-10-107 PMC3108934

[9] Manyando C, Njunju EM, Chileshe J, Siziya S, Shiff C Rapid diagnostic tests for malaria and health workers’ adherence to test results at health facilities in Zambia. Malar J 2014; 13:166. 24885996 10.1186/1475-2875-13-166 PMC4026818

[10] Mukanga D, Tibenderana JK, Kiguli J et al Community acceptability of use of rapid diagnostic tests for malaria by community health workers in Uganda. Malar J 2010; 9:203. 20626863 10.1186/1475-2875-9-203 PMC2914066

[11] Baiden F, Owusu-Agyei S, Okyere E et al Acceptability of rapid diagnostic test-based management of malaria among caregivers of under-five children in rural Ghana. PLoS One 2012; 7:e45556. 23029094 10.1371/journal.pone.0045556 PMC3445487

[12] Mubi M, Janson A, Warsame M et al Malaria rapid testing by community health workers is effective and safe for targeting malaria treatment: randomised cross-over trial in Tanzania. PLoS One 2011; 6:e19753. 21750697 10.1371/journal.pone.0019753 PMC3130036

[13] Phiri TB, Kaunda-Khangamwa BN, Bauleni A et al Feasibility, acceptability and impact of integrating malaria rapid diagnostic tests and pre-referral rectal artesunate into the integrated community case management programme. A pilot study in Mchinji district, Malawi. Malar J 2016; 15:177. 27000034 10.1186/s12936-016-1237-2 PMC4802711

[14] Mukanga D, Tiono AB, Anyorigiya T et al Integrated community case management of fever in children under five using rapid diagnostic tests and respiratory rate counting: a multi-country cluster randomized trial. Am J Trop Med Hyg 2012; 87:21–9. 23136274 10.4269/ajtmh.2012.11-0816 PMC3748518

[15] World Health Organization. World malaria report 2015. Geneva, Switzerland: WHO, 2015.

[16] Newton S, Doku V, Geissler W, Asante KP, Cousens S Drawing blood from young children: lessons learned from a trial in Ghana. Trans R Soc Trop Med Hyg 2009; 103:497–9. 19155032 10.1016/j.trstmh.2008.11.030

[17] Ajayi IO, Nsungwa-Sabiiti J, Siribié M et al Feasibility of malaria diagnosis and management in Burkina Faso, Nigeria, and Uganda: a community-based observational study. Clin Infect Dis 2016; 63 suppl 5:S245–55. 10.1093/cid/ciw622 PMC5146694 27941101

[18] World Health Organization. Severe malaria. Trop Med Int Heal 2014; 19 (suppl 1):7–131. 10.1111/tmi.12313_2 25214480

[19] Okwundu CI, Nagpal S, Musekiwa A, Sinclair D Home- or community-based programmes for treating malaria. Cochrane Database Syst Rev 2013; 5:CD009527. 10.1002/14651858.CD009527.pub2 PMC6532579 23728693

[20] Ajayi IO, Browne EN, Garshong B et al Feasibility and acceptability of artemisinin-based combination therapy for the home management of malaria in four African sites. Malar J 2008; 7:6. 18182114 10.1186/1475-2875-7-6 PMC2254635

[21] Jegede A, Adejumo P, Ushie BA Factors influencing motivation and retention of primary healthcare workers in the rural areas of Oyo State, Nigeria. World Health Popul 2013; 14:23–36. 24289967 10.12927/whp.2013.23580

